# Post-mortem viral dynamics and tropism in COVID-19 patients in correlation with organ damage

**DOI:** 10.1007/s00428-020-02903-8

**Published:** 2020-08-20

**Authors:** Kristijan Skok, Evelyn Stelzl, Michael Trauner, Harald H. Kessler, Sigurd F. Lax

**Affiliations:** 1 Department of Pathology, Hospital Graz II, Academic Teaching Hospital of the Medical University of Graz, Goestingerstrasse 22, AT-8020 Graz, Austria; 2 Diagnostic & Research Institute of Hygiene, Microbiology and Environmental Medicine, Medical University of Graz, Neue Stiftingtalstrasse 6, AT-8010 Graz, Austria; 3 Division of Gastroenterology and Hepatology with Intensive Care 13H1, Department of Internal Medicine III, Vienna General Hospital, Medical University of Vienna, Waehringerguertel 18-20, AT-1090 Vienna, Austria; 4 School of Medicine, Clinical Institute of Pathology and Molecular Pathology, Johannes Kepler University Linz, Huemerstrasse 3-5, AT-4020 Linz, Austria

**Keywords:** COVID-19, SARS-CoV-2, Post-mortem, PCR, Immunohistochemistry bowel

## Abstract

The persistence of SARS-CoV-2 after death of infected individuals is unclear. The aim of this study was to investigate the presence of SARS-CoV-2 RNA in different organs in correlation with tissue damage and post-mortem viral dynamics in COVID-19 deceased. Twenty-eight patients (17 males, 11 females; age 66–96 years; mean 82.9, median 82.5 years) diagnosed with COVID-19 were studied. Swabs were taken post-mortem during autopsy (*N* = 19) from the throat, both lungs, intestine, gallbladder, and brain or without autopsy (*N* = 9) only from the throat. Selective amplification of target nucleic acid from the samples was achieved by using primers for ORF1a/b non-structural region and the structural protein envelope E-gene of the virus. The results of 125 post-mortem and 47 ante-mortem swabs were presented as cycle threshold (Ct) values and categorized as strong, moderate, and weak. Viral RNA was detected more frequently in the lungs and throat than in the intestine. Blood, bile, and the brain were negative. Consecutive throat swabs were positive up to 128 h after death without significant increase of Ct values. All lungs showed diffuse alveolar damage, thrombosis, and infarction and less frequently bronchopneumonia irrespective of Ct values. In 30% the intestine revealed focal ischemic changes. Nucleocapsid protein of SARS-CoV-2 was detected by immunohistochemistry in bronchial and intestinal epithelium, bronchial glands, and pneumocytes. In conclusion, viral RNA is still present several days after death, most frequently in the respiratory tract and associated with severe and fatal organ damage. Potential infectivity cannot be ruled out post-mortem.

**Electronic supplementary material:**

The online version of this article (10.1007/s00428-020-02903-8) contains supplementary material, which is available to authorized users.

## Introduction

The coronavirus disease 2019 (COVID-19) caused by the severe acute respiratory syndrome (SARS)-coronavirus-2 (CoV-2) has become a pandemic and shaken economy, societies, and national healthcare systems worldwide [1]. SARS-CoV-2 has been categorized into the ACDP hazard group 3, which means that (1) the hazard may lead to severe human disease and can be a significant risk to laboratory employees, (2) the virus can be transmitted to other people, and (3) prophylaxis and/or treatment are generally accessible [2]. The currently established potential transmission route is person-to-person, mainly via respiratory aerosols and droplets when an infected person coughs, sneezes, sings, shouts, or talks [3]. The risk of transmission depends on several factors such as the type and duration of exposure, the use of preventive measures, and individual factors (e.g., the amount of virus in respiratory secretions) [4]. Virus-contaminated surfaces can also be a potential source of infection including transmission of the virus to the mucous membranes of the individual, in particular the nose, eyes, and mouth [4, 5]. Infections in healthcare units, including long-term care facilities, are of utmost concern [6, 7]. The infectivity of a person also remains unclear. The virus can be detected in various clinical samples, depending on the severity of symptoms in pharyngeal swabs up to 42 days [8, 9]. In addition to the respiratory tract, the gastrointestinal tract has received increasing attention as site of viral replication and shedding via feces [10, 11].

Post-mortem examinations provide important insights for the study of novel diseases and the clarification of unknown causes of death and are considered an integral part of a quality-assessed healthcare system. Especially in the case of infectious diseases, the safety protection for the examination team is crucial and requires profound knowledge of the potential virulence of an infectious agent. Therefore, it is important to determine whether SARS-CoV-2 is capable to replicate after death of the infected individual and thereby potentially may cause an infection. Pathologists may fear infection while performing autopsies and may therefore avoid them, thus precluding urgently needed pathomechanistic insights into a hereto poorly understood disease.

There is a number of studies dealing with viral dynamics of SARS-CoV-2, but to the best of our knowledge, no study has analyzed the post-mortem setting in correlation with additional ante-mortem reference points. The aim of this study was to determine the temporal-spatial distribution of SARS-CoV-2 in post-mortem swabs of COVID-19 deceased. We used a technically feasible and robust semi-quantitative approach which is widely used for the assessment of the potential infectivity of an individual and further correlated the load of viral RNA with changes in lungs and bowel as frequently involved organs.

## Materials and methods

### Patients characteristics

This prospective study was carried out at the Department of Pathology of the Hospital Graz II, an academic teaching hospital of the Medical University of Graz and the Molecular Diagnostics Laboratory of the Medical University of Graz. From March 24, 2020, to May 13, 2020, 68 patients aged from 66 to 99 (30 female, 38 males, mean 82.91, median 82) died due to COVID-19 in our hospital. Twenty-eight deceased COVID-19 patients were included of which 19 underwent autopsy. All included patients fulfilled the WHO criteria for COVID-19 (www.who.int). They presented with fever and acute respiratory symptoms including cough and shortness of breath and were tested positively for SARS-CoV-2 RNA by PCR either before or at admission to our hospital. The selection of these patients was performed at random and only influenced by our infrastructural, time, and personnel constraints under the challenging pandemic situation. There were no medical exclusion criteria. All deceased patients were brought from the ward to a morgue with a standardized ambient temperature of 4 °C within 2 h after death. Eight of the 19 autopsy cases were included in a previous study, which focused on the clinicopathological findings and did not investigate the viral load of the patients [12].

### Post-mortem sampling

Swabs from other anatomic regions than the pharynx could only be taken from the patients who underwent autopsy. All swabs were taken by the same investigator (K.S.). To investigate the presence of viral RNA after death, the autopsy procedure included post-mortem swabs from the throat and other organs (both the lungs, colon, small intestine, gallbladder, cerebrospinal fluid/brain, and blood). The lung swabs were taken separately from each side of the bronchial system. The swabs from the bowel were taken from the mucosa mostly from the sigmoid colon after incision of the bowel wall using a fresh scalpel. The cerebrospinal fluid/brain swab was taken from the lateral ventricles by crossing the corpus callosum. To avoid cross-contamination, fresh scissors or scalpels were used whenever required. Consecutive throat swabs after autopsy at a 24-h interval were taken from the nasopharynx. In addition, consecutive post-mortem throat swabs were taken from 9 deceased COVID-19 patients who did not undergo autopsy. Results of ante-mortem swabs were retrieved for 25 patients from the database of the Laboratory of Molecular Diagnostics of the Medical University of Graz; for 3 patients, the analysis had been performed in an outside lab, and Ct values were not available. All performed procedures and investigations were in accordance with the ethical standards of the institutional research committee and with the 1964 Helsinki declaration and its later amendments. According to federal Austrian law for public hospitals, autopsies are mandatory without informed consent from the relatives due to scientific or public interest, particularly, if the cause of death is uncertain.

### Viral RNA analysis, correlation with pathological findings, and demonstration of intracellular viral protein by immunohistochemistry

The sampling was done according to the same protocol as described in our previous study [12]. Swabs were collected by using Copan ESwab™ collection system containing 1 mL of transport medium and stored at 2–8 °C until transported to the Molecular Diagnostics Laboratory, Medical University of Graz. Samples were tested for SARS-CoV-2 RNA within 12 h of arrival. The presence of SARS-CoV-2 RNA was determined by real-time PCR (qPCR) using the in vitro diagnostics/Conformité Européenne (IVD/CE)-labeled Cobas® SARS-CoV-2 test (Roche Molecular Systems, Branchburg, NJ, USA) for use on the Cobas® 6800/8800 system (Roche Molecular Diagnostics, Rotkreuz, Switzerland) [13, 14]. Selective amplification of target nucleic acid from the sample was achieved by the use of target-specific forward and reverse primers for ORF1a/b non-structural region that is unique to SARS-CoV-2. In addition, a conserved region in the structural protein envelope E-gene was chosen for pan-sarbecovirus detection. The pan-sarbecovirus detection set also detects SARS-CoV-2 virus.

The results were presented as cycle threshold (Ct) values. The Ct is defined as the number of cycles required for the fluorescent signal to cross the threshold (i.e., exceeds background level). Ct levels are inversely proportional to the amount of target nucleic acid in the sample (i.e., the lower the Ct level, the greater the amount of target nucleic acid in the sample). The Ct values obtained by qPCR were sorted, grouped into 3 categories (< 25, strongly positive; 25–35, moderately positive; > 35 weakly positive) and statistically analyzed.

The viral data from the lungs and the colon were correlated with the organ damage as determined by histopathological examination. The technique for grossing and sampling of the lungs has been previously detailed [12]. In brief, both lungs were totally fixed in formalin and sliced after fixation from the apex to the basis. Multiple samples were processed from each lobe, partially using giant sections. The colon was sampled from the area where the swab was taken. The diffuse alveolar damage was categorized according to stage [15, 16], and the severity of lung damage was appreciated based on the extent of inflammatory and vascular changes and graded using a 3-tiered scale (mild, moderate, severe). We considered focal inflammatory changes in the absence of infarction as mild, extensive inflammation with thrombosis and/or infarction involving at least large parts of a lobe as severe and all changes in between as moderate.

To demonstrate the virus in situ, samples from the lungs and the colon of one representative RNA-positive case were analyzed by immunohistochemistry using a mouse monoclonal antibody against the SARS-CoV-2 nucleocapsid protein (clone 4B21, Creative Diagnostics, Shirley, NY, USA). The analysis was performed on a Ventana Benchmark Ultra™ system (Roche Diagnostics, Indianapolis, IN, USA) with a concentration of 1:250 for the primary antibody. A brown intracytoplasmic reaction product was considered positive. For negative controls, tissue from non-COVID-19 cases was used.

Statistical analysis and presentation of results were performed using Microsoft Excel Office 365 (Microsoft), GraphPad Prism version 8.0 (GraphPad Software, La Jolla California USA) and SPSS Statistics for Windows, version 25.0 (IBM Corp., Armonk, NY).

## Results

All patients were Caucasians, 17 males and 11 females (age range 66–96, mean 82.9, median 82.5 years). The time from onset of symptoms to death took from 4 to 36 days (mean 12.3 days) and the duration of hospitalization from 4 to 35 days (mean 11 days). A total of 125 swabs were collected prospectively post-mortem from the throat (*N* = 57), lungs (*N* = 38; 19 from each side), colon (*N* = 13), cerebrospinal fluid and brain (*N* = 7), gallbladder (*N* = 4), small intestine (*N* = 2), and blood (*N* = 4) at 24-h intervals. Data from 47 ante-mortem throat swabs (before and during hospitalization) were available for 25 patients. The number of post-mortem swabs from one patient ranged from 1 to 7; the number of ante-mortem swabs ranged from 1 to 10.

### Analysis of the samples

**Fig. 1:**
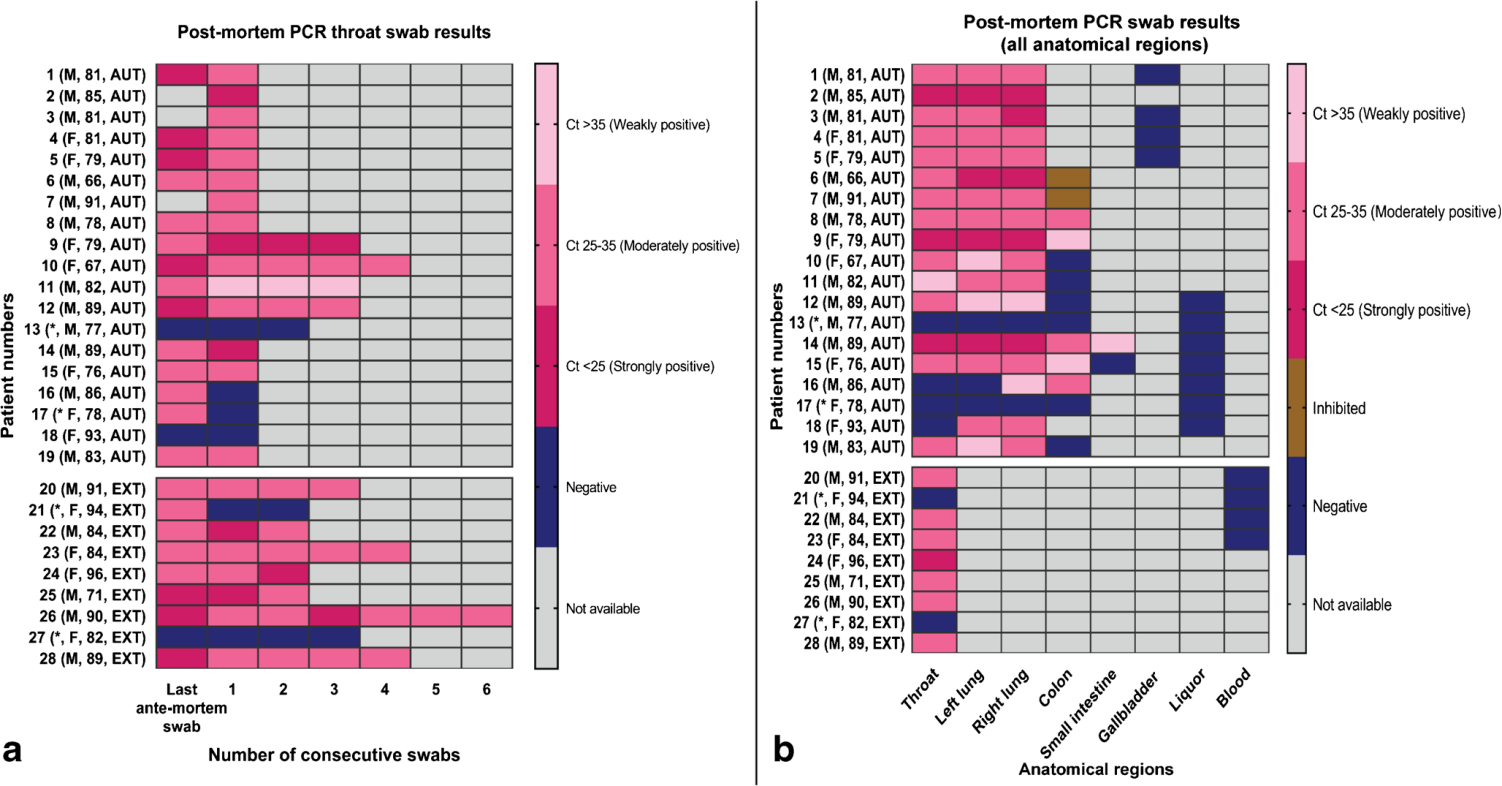
Distribution of qPCR results (according to Ct values) of the collected samples. The post-mortem swab qPCR results from throat (**a**) and all organs (**b**) are shown as heatmaps. qPCR results are grouped as weakly, moderately and strongly positive, negative, and inhibited. M, male; F, female; AUT, autopsy; EXT, external examination. *Patients with exclusively negative post-mortem results

For 22 patients, the initial post-mortem swabs were positive; for the remaining 6 patients, all post-mortem swabs were negative, and in 3 of these 6 patients also, the last ante-mortem swab had been negative. Subsequent post-mortem swabs were taken in 14 patients, and the results of the first post-mortem swab remained positive (11 patients) or negative (3 patients). In 9 of these, 14 patients at least 3 swabs were taken (8 positive), and in 4 of the 14 patients, at least 4 swabs were taken (all 4 positive). The result of the first post-mortem swab remained either positive or negative throughout all consecutive swabs. A switch from a positive ante-mortem to a negative post-mortem throat swab was found in 3 patients (2 underwent autopsy) of whom one had positive post-mortem swabs from the right lung and the colon. In 3 cases, 2 with autopsy, the last ante-mortem and all post-mortem throat swabs were negative. The results are detailed on Fig. 1a.

The anatomical distribution is demonstrated on Fig. 1b. Lung swabs were positive in 17/19 (right lung; average Ct value 28.53) and 16/19 (left lung; average Ct value 29.01) cases, respectively. Most lung swabs fell into the strongly or moderately positive category, whereas none of the intestinal swabs was strongly positive. Swabs from the colon were positive in 5/13 cases (average Ct value 32.44) and from the small intestine in 1/2 cases (average Ct value 35.75). In 2 colon swabs, the PCR reaction was inhibited which means that the detection of the internal control failed due to inhibitory agents such as feces, bile, salts, or complex polysaccharides. All swabs from blood, gallbladder, and cerebrospinal fluid/brain tissue were negative. Due to several negative results, we analyzed only a limited number of samples from blood and the gallbladder.

**Fig. 2:**
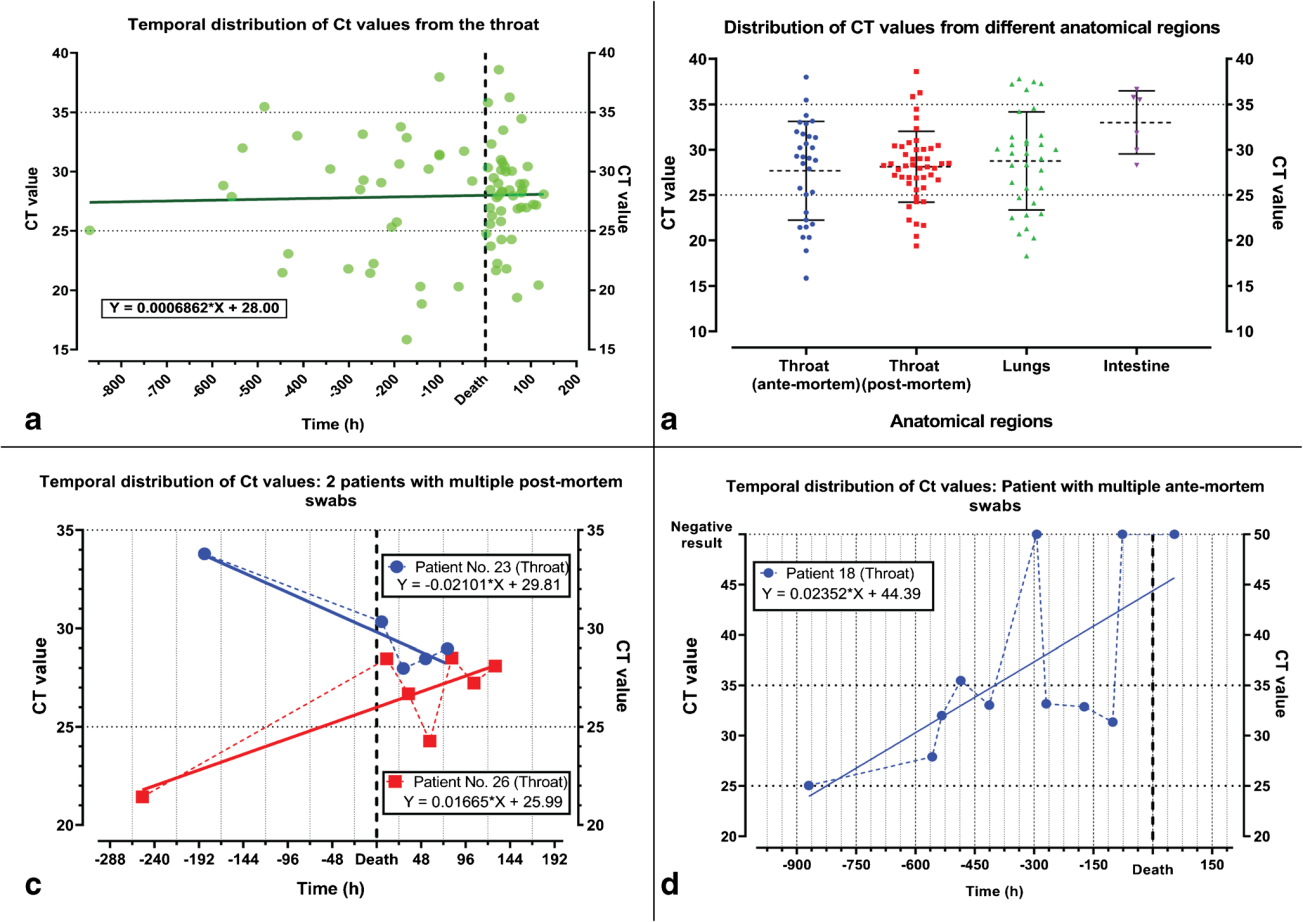
Temporal distribution of Ct values of all pharyngeal swabs from 24 post-mortem positive patients (**a**) and numeric distribution of all swabs (**b**). Temporal distribution of the Ct values in two patients with multiple post-mortem swabs (**c**) and in a patient with 10 ante-mortem and one post-mortem swabs (**d**)

The Ct values for the throat swabs reveal a trend to a slight but not statistically significant increase from the ante-mortem to the post-mortem period (average Ct value 28.12 post-mortem compared with 27.68 ante-mortem) (Fig. 2a). The Ct values for the individual anatomical regions are distributed among all 3 semi-quantitative categories with a predominance of moderate positivity (Fig. 2b). Subsequent swabs from one patient may show a variation of the Ct values over time as demonstrated for 2 cases (Fig. 2c). The variability of Ct values with intermittent negativity is showed in another patient with multiple swabs over 36 days of hospitalization (Fig. 2d). Neither the differences of Ct values from the throat (*M* = 28.5713, SD = 5.11) and from the lungs (*M* = 28.32, SD = 5.26) were statistically significant (*t* (14) = 0.18; *p* = 0.548; paired samples *t* test) nor of throat swabs before (*M* = 27.68, SD = 5.44) and after death (*M* = 28.12, SD = 3.92) (*t* (− 0.4) = 76; p = 0.548; independent sample *t* test).

### Correlation of Ct values with degree of organ damage

**Fig. 3:**
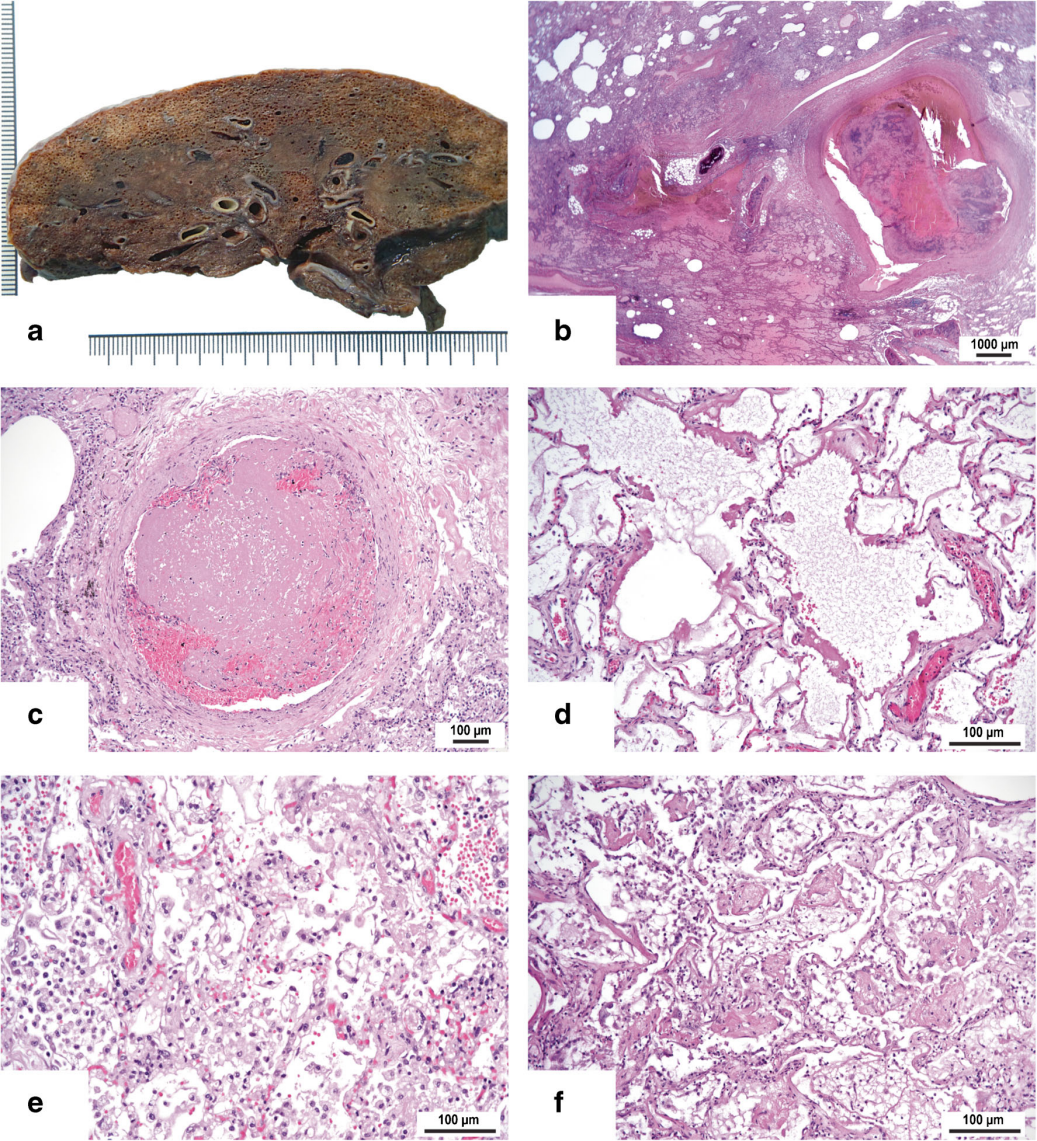
Cross section through the inferior lobe with congestion, thrombotic material in multiple arteries, and induration of the lung tissue (**a**). Hemorrhagic infarction of lung tissue adjacent to a mid-sized pulmonary artery with thrombotic material (**b**). Occlusion of a small artery by a thrombus (“microthrombus”) without infarction of the surrounding lung tissue (**c**). Different stages of diffuse alveolar damage with hyaline membranes and edema (**d**), proliferation of alveolar macrophages with cellular atypia (**e**), and proliferation of fibrous tissue with organizing pneumonia-like pattern (**f**). HE (**b**–**f**), original magnifications × 10 (**b**), × 100 (**c**), and × 200 (**d**–**f**)

The pathological changes of the lungs confirmed our previous findings [12]. Macroscopically, both lungs showed massive congestion in the dorsobasal segments (hypostasis); the pleura was inconspicuous in most cases. The most striking features are thrombotic occlusions of small- to mid-sized pulmonary arteries which were grossly visible (Fig. 3a) and histologically confirmed and often associated with infarction and infarction-like changes (Fig. 3a, b). Histologically, we also found thrombosis of smaller arteries with less than 1 mm in diameter (Fig. 3c). In all cases, the lung parenchyma showed a diffuse alveolar damage (DAD) at various stages and in varying degrees characterized by edema and hyaline membranes (acute or exudative phase), proliferation of pneumocytes and fibroblasts with organization of the hyaline membranes (subacute or organizing phase), and in some cases also interstitial fibrosis and organizing pneumonia (fibrotic or chronic phase) (Fig. 3d–f). In a subset of cases, the proliferative phase was associated with reactive atypical changes of the pneumocytes and squamous metaplasia. Three quarters of the cases showed mostly focal bronchopneumonia mostly associated with purulent bronchitis. The degree of organ damage was severe in 12 cases and moderate in 7 cases. The pathological findings are detailed in Table 1.

**Table 1:** Clinical and viral data of 19 patients with autopsy in correlation with organ damage

Patient number	1	2	3	4	5	6	7	8	9	10	11	12	13	14	15	16	17	18	19
Clinical data																			
Age	81	85	81	81	79	66	91	78	79	67	82	89	77	89	76	86	78	93	83
Gender	M	M	M	F	F	M	M	M	F	F	M	M	M	M	F	M	F	F	M
Onset of symptoms to death (days)	11	11	10	4	6	5	10	18	8	20	8	13	15	9	14	9	36	36	12
Duration of hospitalization (days)	7	6	8	4	5	6	9	11	8	20	8	12	14	8	13	8	35	34	11
Ct values of swabs																			
Throat: First a.m.	15.84	Pos., Ct nav	Pos., Ct nav	18.85	20.33	30.22	Pos., Ct nav	21.47	25.75	22.24	30.22	23.07	32.8	29.07	37.97	28.83	26.55	25.04	31.73
Throat: Last a.m.	Same	Pos., Ct nav	Pos., Ct nav	Same	Same	Same	Pos., Ct nav	31.46	Same	Same	Same	Same	Neg	Same	29.21	Same	33.41	Neg	Same
Throat: First p.m.	33.5	22.25	34.47	29.02	28.26	28.21	30	30.43	21.65	29.48	35.83	26.95	Neg	20.44	26.98	Neg	Neg	Neg	30.13
Right lung	26.39	22.94	24.7	29.04	30.56	22.79	25.76	31.59	21.27	30.03	30.99	37.21	Neg	18.28	34.21	37.26	Neg	31.41	30.55
Left lung	25.80	24.12	29.62	29.61	28.35	22.49	27.76	30.08	20.70	36.60	28.29	37.46	Neg	20.25	34.57	Neg	Neg	30.68	37.79
Colon	NA	NA	NA	NA	NA	Inhib	Inhib	29.95	35.51	Neg	Neg	Neg	Neg	31.84	36.63	28.29	Neg	NA	Neg
Organ damage																			
Lungs																			
Edema	Yes	Yes	Yes	Yes	Yes	Yes	Yes	Yes	Yes	Yes	Yes	Yes	Yes	Yes	Yes	Yes	Yes	Yes	Yes
Hyaline membranes	Yes	Yes	No	Yes	Yes	Yes	Yes	Yes	Yes	Yes	Yes	Yes	Yes	Yes	Yes	Yes	Yes	Yes	Yes
Proliferation	Yes	Yes	No	Yes	Yes	Yes	Yes	Yes	Yes	Yes	Yes	Yes	Yes	Yes	Yes	Yes	Yes	Yes	Yes
Pulmonary artery thrombosis	Yes	Yes	Yes	Yes	Yes	Yes	Yes	Yes	Yes	Yes	Yes	Yes	Yes	Yes	Yes	Yes	Yes	Yes	Yes
Infarction	Yes	Yes	Yes	No	No	Yes	Yes	Yes	Yes	Yes	Yes	Yes	Yes	Yes	No	Yes	Yes	Yes	Yes
Bronchopneumonia	No	Yes	Yes	No	Yes	No	Yes	Yes	Yes	Yes	No	Yes	Yes	Yes	Yes	Yes	Yes	Yes	Yes
Fibrosis	No	Yes	Yes	No	Yes	No	Yes	Yes	No	Yes	Yes	Yes	Yes	No	Yes	Yes	Yes	Yes	Yes
Degree of organ damage	Sev	Sev	Mod	Mod	Mod	Sev	Sev	Sev	Sev	Sev	Sev	Sev	Sev	Mod	Mod	Mod	Sev	Mod	Sev
Bowel																			
Ischemic changes	No	No	No	No	No	No	No	Yes	No	No	No	Yes	Yes	Yes	No	No	Yes	Yes	No

*a.m.* ante mortem; *p.m.* post-mortem; *NA* not assessed; *Neg* negative; *Inhib* inhibited (detection of the internal control failed due to inhibitory agents); Pos*. Ct nav* test positive but Ct values were not available (tests were performed in other labs); *Same* data from only one ante-mortem swab were available; *Sev* severe; *Mod* moderate; *M* male; *F* female

**Fig. 4:**
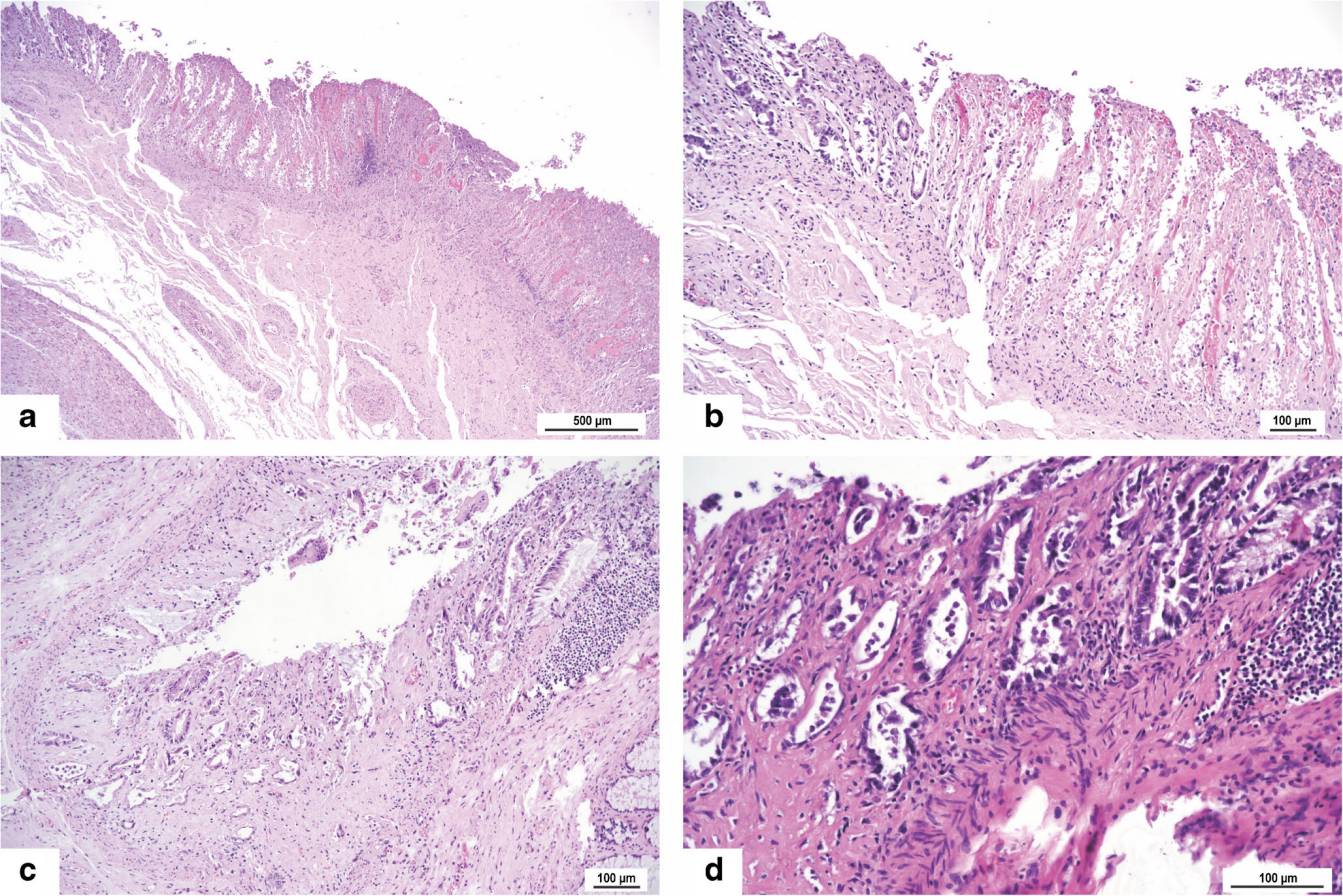
Ischemic colopathy of different extent but limited to the mucosa: Necrosis and hemorrhagic (**a**, **b**) and atrophic crypts with cryptitis containing neutrophils (**c**, **d**). HE, original magnifications × 40 (**a**), × 100 (**b**, **c**), and × 200 (**d**)

The bowel was grossly inconspicuous in all cases including the peritoneal surface but on histological examination revealed focal ischemic changes limited to the mucosa in 6 cases. The ischemic changes were characterized by atrophic crypts, cryptitis, ulceration, and hemorrhage (Fig. 4). Except for one case, they were present in patients with long duration of disease. No other pathological changes were found.

**Fig. 5:**
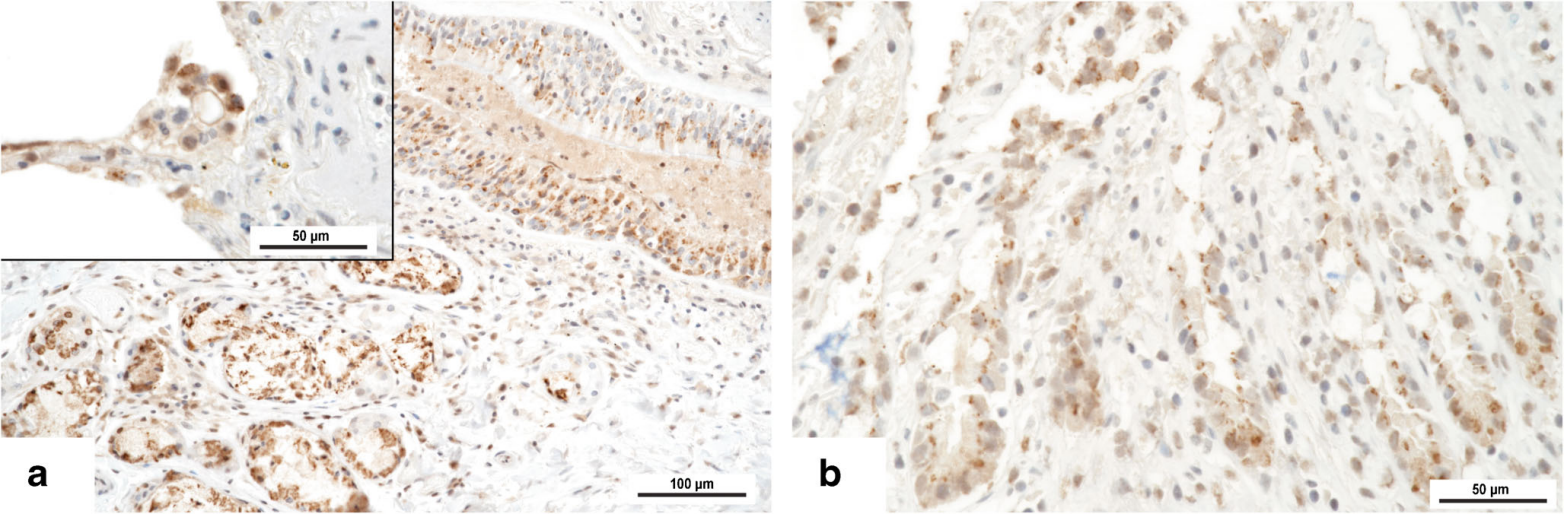
SARS-CoV-2 nucleocapsid protein in bronchial epithelium, bronchial mucus glands, and pneumocytes (insert) (**a**) and in intestinal epithelium of the crypts (**b**). DAB, original magnifications × 200 (**a**, **b**) and × 400 (insert of **a**)

The individual Ct values did not correlate with the severity of organ damage. However, Ct values were lower in the lungs reflecting a higher RNA load compared with the intestines, and the lungs were significantly more often positive than the bowel (*p* = 0.028; chi^2^ test with Yates correction). Notably, the degree of organ damage was moderate or severe in the lungs in all cases, whereas it was mild and focal in the bowel and present only in about 30% of the cases. The Ct values for the non-autopsy cases were correlated with the duration of disease and hospitalization, respectively, which did not reveal statistical significance (supplementary table). Viral nucleocapsid protein could be demonstrated by immunohistochemistry in the respiratory epithelium and in the mucous glands of the bronchi as well as in pneumocytes (Fig. 5a). In the colon, the viral protein was detected in the surface and crypt epithelium (Fig. 5b).

## Discussion

In this study, we performed consecutive post-mortem sampling from the pharynx and found viral RNA up to 128 h after death with only a slow decrease of positivity. We further demonstrate that the throat and lungs are most frequently affected irrespective of the positivity of the pharyngeal swab and show only slight differences of the average Ct values. This is in contrast to a recent autopsy study which reported clearly higher viral load in the lungs compared with the pharynx by using a different sampling method [17]. Our findings further suggest that viral RNA is less frequently found in intestinal tissue compared with the respiratory tract and the distribution between the two sites varies. This could be demonstrated in four cases with negative intestinal swabs but positive throat and lung swabs. On the other hand, we were able to detect viral RNA in the colon in one case where swabs from the throat and the left lung were negative. This is in line with recent reports on positive fecal viral RNA in the absence of positive nasopharyngeal swabs [10, 11, 18]. Previous reports suggest that SARS-CoV-2 RNA is significantly longer to be detected in stool samples than in samples from the respiratory tract [19]. Viral organotropism in our study is discrepant to some clinical studies, which report viral RNA in conjunctival swabs, blood samples, gastric juice, feces, anal swab, and urine [20–23]. In this study, we neither found viral RNA in swabs from blood nor in swabs from the brain and cerebrospinal fluid, but the number of analyzed samples from these anatomical areas was limited. Viral RNA was detected at low levels in brain tissue and blood samples by another autopsy study which, in contrast to us, performed viral analysis by qPCR on homogenized frozen tissue and in addition in situ hybridization and immunofluorescence on formalin-fixed, paraffin-embedded tissue [17].

The degree of organ damage does not show any correlation with the Ct values, neither within one organ nor between different organs. In particular, pathological changes of the lungs are by far more severe and extensive than the focal ischemic alterations of the intestines. In the lungs, both the diffuse alveolar damage involving the parenchyma and the vascular occlusions by thrombotic material contribute to the massive cardiorespiratory failure and terminal breakdown [12, 24]. It is likely that the pathomechanisms induced by the SARS-CoV-2 infection and leading to organ damage are different between the lung and intestines and are responsible for distinct patterns of response. The lung parenchyma seems to respond to SARS-CoV-2 infection by a similar mechanism compared with shock, toxins, and other viral agents which causing acute lung injury. Diffuse alveolar damage, the histological substrate of acute respiratory distress syndrome (ARDS), belongs to its spectrum [25, 26]. Obviously, the formation of hyaline membranes is caused by a direct injury of the cells lining the blood-air barrier, in particular endothelial cells and pneumocytes through the virus and a breakdown of the barrier [25]. In a recent study, viral protein was massively detected in pneumocytes besides the bronchial epithelium and the bronchial mucosal glands [27], which we could also demonstrate. It is unclear whether the subsequent stages of diffuse alveolar damage including the arterial thrombosis are stimulated and propagated by the virus or whether their severity depends mainly on the degree of tissue alteration by the initial infection as “first hit.” On the other hand, the pathological changes of the bowel seem to be limited to ischemic changes (“ischemic colitis”) with focal, often microscopic mucosal lesions. They might rather reflect multiorgan failure due to breakdown of the systemic circulation than direct viral injury although viral protein could be detected within the colon epithelium by immunohistochemistry.

The temporal dynamics of SARS-CoV-2 are crucial for its potential virulence particularly in the post-mortem setting. This has been investigated previously for SARS-CoV-1 [28]. In this study, we used Ct values for a semi-quantitative approach to assess and compared the viral load over a certain time period. If viral RNA was detectable within 24-h post-mortem, it remained detectable without a significant increase of the Ct value. On the other hand, if viral RNA was not detectable within 24-h post-mortem, the case remained negative. We are aware that the Ct values do not correspond with infectivity, although high Ct values represent a low viral RNA load and harbor a lower probability of transmission of infection. In fact, infectivity could only be determined using viral cultures [29], but this was unfortunately beyond the scope of the current study. Recently, vital virus could be transferred from lung samples from autopsies performed 6-day post-mortem and cultivated in cell cultures but without reporting Ct values [30]. On the other hand, a recent correlation of Ct values with successful isolation of virus in cell cultures suggested that patients with Ct values above 33–34 were no longer contagious [31]. Furthermore, in a study of nine patients with mild COVID-19, infectious virus was not detected from respiratory specimens when the viral RNA level was < 10^6^ copies/mL [29]. This information might be considered for discontinuation of isolation in patients recovering from COVID-19 and for post-mortem examination and autopsies but lacks certainty and requires confirmation. In addition, the methodology of various studies has been heterogenous and inconsistent [32].

We are aware that our study has certain limitations, particularly, by the small number of included cases, the varying number of tested samples among the different cases, and the semi-quantitative method. The varying number of samples was particularly influenced by the time the deceased stayed in our morgue. Viral culture was beyond the scope of the current study; in addition, it is laborious, not widely available, and requires several weeks to obtain results. Further studies will have to explore the infectious potential of RNA-positive samples post-mortem. Nevertheless, to the best of our knowledge, this is the first study that shows the temporal dynamics of SARS-CoV-2 in the post-mortem setting including a correlation with ante-mortem data.

In conclusion, the severity of parenchymatous and vascular pulmonary changes in fatal COVID-19 cannot be explained simply by the degree of the viral RNA load in the respiratory system. Further studies will have to explore the role of other mechanisms including an overwhelming host immune reaction. Finally, our findings also suggest the importance of post-mortem testing before autopsy and of careful safety procedures since viral RNA may be still detectable and despite moderately to weakly positive Ct values a potential virulence cannot be ruled out.

## Electronic supplementary material

ESM 1 (DOCX 14 kb)
