# Supplementary material for: Post-mortem viral dynamics and tropism in COVID-19 patients in correlation with organ damage
Source: Virchows Arch. 2020 Aug 20;478(2):343–53. doi: 10.1007/s00428-020-02903-8 (PMC7438212; doi:10.1007/s00428-020-02903-8)
Supplement: Supplementary file 1 — (DOCX 14 kb) [file 428_2020_2903_MOESM1_ESM.docx]

**Supplementary table**: Clinical and viral data of 9 patients without autopsy

| **PATIENT Number** | **20** | **21** | **22** | **23** | **24** | **25** | **26** | **27** | **28** |
| --- | --- | --- | --- | --- | --- | --- | --- | --- | --- |
| **Clinical data** |  |  |  |  |  |  |  |  |  |
| Age | 91 | 94 | 84 | 84 | 96 | 71 | 90 | 82 | 89 |
| Gender | M | F | M | F | F | M | M | F | M |
| Onset of symptoms to death (days) | 13 | 12 | 12 | 5 | 9 | 9 | 10 | 11 | 9 |
| Duration of hospitalisation (days) | 12 | 11 | 9 | 5 | 8 | 8 | 10 | 11 | 8 |
| **CT VALUES of throat swabs** |  |  |  |  |  |  |  |  |  |
| First positive a.m. | 29.28 | 28.14 | 30.65 | 33.79 | 25.32 | 21.79 | 21.43 | 30.91 | 28.48 |
| Last positive a.m. | same | 28.14 | same | same | same | same | same | neg | 20.32 |
| First p.m. | 32.33 | neg | 24.76 | 30.34 | 25.57 | 23.71 | 28.47 | neg | 26.28 |

Legend: a.m. - ante mortem; p.m. - post mortem; neg - negative; same: data from only one ante-mortem swab were available, M – male, F – female.
